# Does Avoiding Distressing Thoughts and Feelings Influence the Relationship between Carer Subjective Burden and Anxiety Symptoms in Family Carers of People with Dementia?

**DOI:** 10.1177/07334648231156858

**Published:** 2023-02-09

**Authors:** Elien Van Hout, Milena L Contreras, Eneida Mioshi, Naoko Kishita

**Affiliations:** 1 School of Health Sciences, University of East Anglia, Norwich, UK

**Keywords:** caregivers, Alzheimer’s disease, psychological inflexibility, anxiety, carer stressors

## Abstract

Anxiety remains understudied in family carers of people with dementia. Understanding factors that moderate the relationship between stressors and anxiety symptoms in this population is critical to inform interventions. This study examined whether generic experiential avoidance (AAQ-II) and experiential avoidance specific to caregiving-related thoughts and feelings (EACQ) moderate the relationship between subjective burden (ZBI-12) and anxiety symptoms (GAD-7) in carers of people with dementia. The first model (*R*^2^ = .66, *∆R*^*2*^ = .03) exploring the moderating effect of AAQ-II demonstrated a significant interaction term between AAQ-II and subjective burden. The second model (*R*^2^ = .53, *∆R*^*2*^ = .03), exploring the moderating effect of EACQ, demonstrated a significant interaction term between EACQ and subjective burden. These results provide evidence that carers with higher levels of experiential avoidance may be particularly prone to the negative effect of subjective burden on anxiety symptoms. Clinical implications for assessment of experiential avoidance and its treatment in carers of people with dementia are discussed.

**What this paper adds**

- This study provides evidence supporting that experiential avoidance may enhance the negative effect of carer subjective burden on anxiety symptoms.
- The strength of this effect seems to increase as levels of carer subjective burden rise.
- There is potential that generic experiential avoidance (measured by the AAQ-II) may have a stronger moderating effect than experiential avoidance specific to caregiving-related thoughts and feelings (measured by the EACQ).

**Applications of study findings**

- Early interventions to undermine experiential avoidance may be beneficial for carers of people with dementia.
- Monitoring the outcome of such interventions using the AAQ-II as the dementia progresses is recommended.

## Introduction

It is estimated that one in three people will care for a person with dementia in their lifetime (National Collaborating Centre for Mental Health, 2018). Due to an increase in life expectancy and prevalence of dementia, these numbers are likely to increase in the next decade (Alzheimer’s Society, 2014). Supporting someone with dementia is known to have a significant negative impact on the wellbeing of family carers (Ali & Bokharey, 2016). Particularly, the prevalence of anxiety is considered to be high among this population with approximately one-third of family carers presenting clinical levels of anxiety symptoms (Kaddour & Kishita, 2020). This prevalence rate is greater than that of family carers of people with other conditions, such as cancer (Friðriksdóttir et al., 2011) and stroke (Loh et al., 2017).

Nevertheless, the current dementia literature mainly focuses on carer depression as an outcome and carer anxiety is somewhat neglected. In addition, existing carer interventions, such as Cognitive Behavioral Therapy (CBT), are considered to be not as effective for anxiety as for depression in family carers of people with dementia (Kishita et al., 2018). Therefore, understanding factors that can moderate the relationship between carer stressors and anxiety symptoms is critical to inform future interventions aimed at improving the wellbeing of family carers of people with dementia.

Carer subjective burden refers to personal appraisals of burden including the physical, psychological, social and emotional impact their caring role has on their life (Liu et al., 2020). A recent meta-analysis, which reviewed 74 studies on informal carers including 24 studies targeting informal carers of people with dementia, demonstrated that carer subjective burden is a key determinant of anxiety symptoms (Del-Pino-Casado et al., 2021). Identifying individual factors that may moderate the association between carer subjective burden and anxiety symptoms could help find a way to prevent family carers experiencing high levels of burden from developing clinically significant anxiety.

Experiential avoidance is the attempt to alter the form, frequency or intensity of private experiences such as thoughts or feelings, even when doing so is costly, ineffective or unnecessary (Hayes et al., 2013). Recently, studies have shown that experiential avoidance is significantly associated with psychological wellbeing among family carers of people with dementia (Cookson et al., 2020; Kishita et al., 2020). In addition, there is considerable evidence suggesting that experiential avoidance moderates the relationship between stressors and mental health outcomes in non-carer populations and higher experiential avoidance has been associated with worse mental health at greater stressor levels (Cookson et al., 2020; Trindade et al., 2020). Therefore, this study aims to investigate the moderating role of experiential avoidance in the relationship between a well-known stressor (carer subjective burden) and anxiety symptoms in family carers of people with dementia.

The most widely used measure of experiential avoidance in research is the Acceptance and Action Questionnaire-II (AAQ-II). The AAQ-II is considered a generic measure of experiential avoidance and has been validated across various populations (Bond et al., 2011; Fledderus et al., 2012). The AAQ-II is a brief measure containing only seven items and thus can be easily administrated in clinical and research settings. However, it has been argued that the AAQ-II may not be sensitive enough for detecting experiential avoidance in particular populations as the items are generic, not targeting domain-specific thoughts and feelings (Hayes et al., 2004).

To overcome the limitation of the AAQ-II, researchers have developed several variations of instruments assessing experiential avoidance that are more specific to certain populations (Ong et al., 2019). These domain-specific measures have the advantage of assessing experiential avoidance directly related to a specific context and may perform better than a generic measure of experiential avoidance, allowing to predict changes in specific behavior more accurately (Schmalz & Murrell, 2010). In this regard, a domain-specific measure of experiential avoidance has been developed for the carer population, the Experiential Avoidance in Caregiving Questionnaire (EACQ; Losada et al., 2014). This study aims to explore the moderating role of experiential avoidance in the relationship between carer subjective burden and anxiety symptoms using both, a generic and a domain-specific measure of experiential avoidance. The findings may inform directions of future research on experiential avoidance in dementia caregiving and provide important clinical implications in terms of assessment and intervention.

Considering the well-established moderating effect of experiential avoidance in non-carer populations between stressors and mental health outcomes, we hypothesize that both generic experiential avoidance and experiential avoidance in caregiving would moderate the relationship between carer subjective burden and anxiety symptoms in family carers of people with dementia. That is, higher levels of experiential avoidance would associate with greater anxiety symptomatology at higher levels of carer subjective burden. Carers who thus report high levels of carer subjective burden and present with high levels of experiential avoidance, are likely to report higher levels of anxiety symptomatology than carers with high levels of carer burden but low levels of experiential avoidance. Considering the possible differences in the moderating effects of general and domain-specific measures of experiential avoidance, we hypothesize that the domain-specific measure of experiential avoidance (EACQ) would account for more variance in anxiety symptoms than the generic measurement of experiential avoidance (AAQ-II).

## Materials and Methods

### Study Design and Sampling

This is a secondary analysis of data from an intervention study assessing the feasibility and acceptability of an online self-help Acceptance and Commitment Therapy (ACT) programme (Kishita et al., 2022). Screening data collected before the intervention phase were used for this study. The recruitment took place between August 2020 and January 2021 in England. Participants were included if they (1) were primary carers; (2) provided regular care to their family member with dementia (i.e., participants were asked whether they had regular contact with the care recipient providing support) and (3) were interested in engaging with an online self-help ACT programme. No criteria were set for frequency of care provide (e.g., hours of caring per week) as the psychological impact of caring is related to multiple factors such as the relationship they have with the person with dementia. We collected background and demographic data such as frequency of care they provide and their relationship to the care recipient instead. Seventy-nine eligible carers were recruited through clinician referrals from three national health services (NHS) through referrals from other ethically approved dementia studies led by the same research team, through self-referral from the community via advertisements in local newspapers, or a national recruitment website (i.e., Join Dementia Research). All participants provided written consent, via post or electronically, before attending the screening session. Full ethical approval was received from the NHS London-Queen Square Research Ethics Committee (20/LO/0025).

### Procedure

Potential participants were contacted by the research team via telephone or email to check their eligibility. Participants meeting the eligibility criteria received an invitation letter and information sheet. Those who provided written consent were then invited to the screening session. Due to the COVID-19 pandemic, an appointment for the screening session was made remotely via video call or telephone. During this screening session, participants were asked to complete self-reported questionnaires using an online survey platform or hardcopies, which were sent home and returned via post, in the remote presence of a researcher.

### Measures

#### Demographic Information

Demographic information including carer age, gender, relationship to the care recipient, and the length of care in months were collected to characterize the sample.

#### Carer Anxiety

The severity of anxiety symptoms was measured using the Generalized Anxiety Disorder Scale (GAD-7; Spitzer et al., 2006). The GAD-7 is a 7-item self-report questionnaire, which assesses how often an individual has experienced anxiety symptoms during the past 2 weeks (e.g., “Over the last 2 weeks, how often have you been bothered by not being able to stop or control worrying?”). Each item is scored on a 4-point scale ranging from 0 (*not at all*) to 3 (*nearly every day*). The total score indicates anxiety severity of minimal (0–4), mild (5–9), moderate (10–14), or severe (15–21). The GAD-7 has good psychometric properties with good internal consistency (Cronbach Alpha = .89) (Spitzer et al., 2006). The Cronbach’s alpha for the current study was .92.

#### Carer Subjective Burden

The 12-item version of the Zarit Burden Interview (ZBI-12; Bédard et al., 2001) was used to assess carer burden. The ZBI-12 assesses two domains of carer subjective burden: personal strain (e.g., “Do you feel you have lost control of your life since your relative’s illness?”) and role strain (e.g., “Do you feel you should be doing more for your relative?”). Each item is scored on a 5-point scale from 0 (*never*) to 4 (*almost always*). The total score ranges from 0 to 48, with higher scores representing higher levels of carer subjective burden. The ZBI-12 has good psychometric properties with good internal consistency (Cronbach Alpha = .88) (Bédard et al., 2001). The Cronbach’s alpha for the current study was .88.

#### Generic Experiential Avoidance

The Acceptance and Action Questionnaire (AAQ-II; Bond et al., 2011) is the most widely used unidimensional measure of experiential avoidance. The AAQ-II is not specifically designed for certain groups or conditions but has been validated across various populations, including non-clinical samples (Bond et al., 2011) and people with mental health problems (Fledderus et al., 2012; Spinhoven et al., 2014). The AAQ-II consists of 7 items rated on a seven-point scale from 1 (*never true*) to 7 (*always true*). The items of the AAQ-II include statements such as “My painful experiences and memories make it difficult for me to live a life that I would value.” The total score ranges from 7 to 49, with higher scores indicating greater levels of experiential avoidance. The AAQ-II has good psychometric properties with good internal consistency (Cronbach Alpha = .88) (Bond et al., 2011). The Cronbach’s alpha for the current study was .93.

#### Experiential Avoidance in Caregiving

The Experiential Avoidance Caregiving Questionnaire (EACQ; Losada et al., 2014) is a 15-item self-reported measure specifically designed to assess experiential avoidance in the caregiving context (the tendency to control, avoid, or suppress distressing thoughts and feelings related to caregiving). The EACQ consists of items related to active avoidant behaviors (e.g., “I tend to ‘ignore’ the negative thoughts that come to me about my relative”), intolerance of negative thoughts and emotions towards the relative (e.g., “I cannot bear it when I get angry with my relative”), and apprehension concerning negative internal experiences related to caregiving (e.g., “It is normal for a carer to have negative thoughts about the person they are caring for”). The original version of the EACQ was developed in Spanish. The English-translated version of the EACQ presented in the original validation study (Losada et al., 2014) has also been used in previous research (George & Ferreira, 2020; Smith et al., 2018). The EACQ is rated on a 5-point scale ranging from 1 (*not at all*) to 5 (*a lot*). The total score ranges from 15 to 75, with higher scores indicating higher levels of experiential avoidance in caregiving. The EACQ has good psychometric properties with acceptable internal consistency (Cronbach Alpha = .70) (Losada et al., 2014). The Cronbach’s alpha for the current study was .73.

### Statistical Analysis

All analyses were performed using IBM SPSS statistical software (Version 28). Data were examined for accuracy with no extreme outliers detected and most variables approximating normality. The percentage of missing data across the eight variables varied between 1.3% and 2.5%. Of the 79 participants, one respondent did not complete the Experiential Avoidance Caregiving Questionnaire (EACQ) while another respondent did not complete both the Zarit Burden Interview (ZBI-12) and the Experiential Avoidance Caregiving Questionnaire (EACQ). This resulted in a dataset of 77 family carers in the moderation model of EACQ and a dataset of 78 family carers in the moderation model of AAQ-II. To ensure consistency among both models, the participant that didn’t complete the EACQ was removed from the final moderation model of AAQ-II. Removing this participant did not result in any difference of significance.

A descriptive analysis of demographic data was performed to categorize the sample. To examine the associations between all variables and account for possible issues of multicollinearity, Pearson’s *r* correlations were conducted between control variables (carer age, gender, relationship to care recipient and length of care) and independent and moderator variables (ZBI, AAQ-II, and EACQ), and the dependent variable (anxiety symptoms). Correlations of *r* < .30 were considered small, *r* ≥ .30–.49 medium or moderate, and *r* ≥ .50 were considered strong (Kraemer et al., 2003). Control, independent and moderator variables that demonstrated significant correlations with anxiety symptoms at *p* < .05 level were included in the moderation analyses. When a correlation coefficient of > .70 among two or more variables is identified, there may be a presence of multicollinearity (Shrestha, 2020). If multicollinearity was identified, the least significant of the multicollinear variables was removed from the models.

A separate moderation analysis was performed for each potential moderator (AAQ-II and EACQ) using the PROCESS computation macro (Model 1; Hayes, 2013) to examine their reverse buffering effect on the link between carer subjective burden (X) and anxiety symptoms (Y). Demographic variables significantly correlated with anxiety symptoms were entered into the model as covariates to control for their potential confounding effects.

All continuous variables that defined the result were mean centered as literature suggests this can have important implications for the interpretability of regression coefficients and multicollinearity within the context of interaction modeling (Shieh, 2011). In addition, a 95% bootstrap resampling procedure (with 5000 samples) was used. Standardized coefficients were calculated (McCabe et al., 2018) and statistical significance was established when the 95% confidence intervals (CIs) did not include zero (Hayes, 2013). In addition, a simple slope analysis was performed using non-standardized coefficients of the continuous variables. Simple slope graphs were plotted for each moderator (AAQ-II and EACQ) with different levels of the moderator (i.e., low, average and high levels of experiential avoidance) to visualize the reverse buffering effects of different types of experiential avoidance. Given that there are no theoretical cut points for both the AAQ-II and the EACQ, this simple slope analysis considered the following cut-point values: one *SD* above the mean, the mean, and one *SD* below the mean (Cohen et al., 2003).

## Results

### Participants

Descriptive statistics demonstrated that the majority of participants were female spouses, who lived with the care recipient in the same household. On average, participants were taking care of their care recipient for 53 months and nearly half of the care recipients were diagnosed with Alzheimer’s disease (43%). Participants’ age ranged from 32 to 85 with 43% of participants being older than 65 years. Forty-two per cent of participants showed minimal symptoms of anxiety, while 39% demonstrated mild symptoms and 8% and 12% of participants demonstrated moderate and severe symptoms, respectively. Other demographic information and means and standard deviations of measurements are shown in Table 1.

**Table 1.** Demographics Variables (*N* = 77).

Carer demographic variable	Percentage or M *(SD)*
Age	63.47 *(10.64)*
Female	73%
Type of relationship with care recipient	
Spousal relationship	52%
Non-spousal relationship	48%
Living with the person with dementia	
Yes	62%
No	38%
Hours of caring per week	
0–2 h	4%
3–10 h	21%
11–20 h	10%
21–40 h	7%
41–80 h	14%
81+h	44%
Length of care (in months)	53.39 *(41.77)*
Anxiety symptoms (GAD-7), score range 0–21	6.45 *(5.27)*
Experiential Avoidance (AAQ-II), score range	20.43 *(9.85)*
Experiential Avoidance in Caregiving (EACQ), score range	41.42 *(8.95)*
Care recipient demographic variables	Percentage or M *(SD)*
Dementia type	
Alzheimer’s	43%
of which early onset Alzheimer’s	1%
Mixed	29%
Vascular	12%
Frontotemporal	3%
Lewy bodies	4%
Unknown	9%

Notes. AAQ-II, Action and Acceptance Questionnaire; EACQ, Experiential Avoidance in Caregiving Questionnaire; GAD-7, Generalized Anxiety Disorder Scale.

### Correlations

A series of Pearson’s *r* correlations were conducted (see Table 2). Carer age was negatively associated with anxiety symptoms, meaning being younger of age was associated with greater anxiety symptoms, and carer gender was positively correlated with anxiety symptoms, meaning female carers were more likely to report anxiety symptoms. Therefore, age and gender were controlled in both moderation analyses. No correlation between the control, independent and moderator variables exceeded the recommended threshold of .70. Issues of multicollinearity were therefore not identified.

**Table 2.** Pearson’s *r* Correlations (*N* = 77).

Variables	1	2	3	4	5	6	7	8	9	10
1. Carer age	1.00									
2. Carer gender	−.38**	1.00								
3. Spousal relationship	−.68**	.30**	1.00							
4. Length of care	.15	−.12	.00	1.00						
5. Living with the person with dementia	.39**	−.36**	−.00	.04	1.00					
6. Hours of caring per week	.10	−.06	−.36**	.11	.64**	1.00				
7. Experiential avoidance	−.24*	.30**	.07	−.11	−.17	.02	1.00			
8. Experiential avoidance in caregiving	−.01	−.07	−.15	−.13	.04	.14	.36**	1.00		
9. Carer subjective burden	−.18	.14	−.10	−.01	.09	.26*	.67**	.31**	1.00	
10. Carer anxiety	−.32**	.29**	−.01	−.17	−.02	.16	.76**	.36**	.63**	1.00

**. Correlation is significant at the .01 level (2-tailed). *. Correlation is significant at the .05 level (2-tailed).

### Moderation Effects

#### Generic experiential Avoidance (AAQ-II)

The unstandardized coefficients (*b*) and standard errors (SE) of the variables (independent variables [carer subjective burden]; moderator [experiential avoidance]; interaction [carer subjective burden X experiential avoidance]; and the two control variables [age and gender]) and the model summary are presented in Table 3.

**Table 3.** Results of Moderation Analysis - Generic experiential avoidance (AAQ-II).

	β	Estimate (B)	SE	*t*	LLCI	ULCI	*p*
Intercept		9.20	3.30	2.78	2.61	15.79	**.007**
ZBI_centered (*X*)	.27	.16	.06	2.82	.05	.27	**.006**
AAQ-II_centered (*W*)	.44	.24	.06	4.09	.12	.35	**<.001**
ZBI x AAQ-II (*X* x *W*)	.21	.01	.00	2.65	.00	.02	**.010**
Age (C_1_)	−.14	−.07	.04	−1.82	−.14	.01	.521
Gender (C_2_)	.05	.59	.92	.65	−1.23	2.42	.073
							
					*R*^2^ = .66, *MSE* = 10.22		
					*F* (5,71) = 27.15, *p* < .001		

*Notes.* ZBI, Zarit Burden Interview [assessment of carer subjective burden]; AAQ, Action and Acceptance Questionnaire [broad assessment of experiential avoidance]; LLCI, Lower level of 95% confidence interval for Estimate (B); ULCI, Upper level of 95% confidence interval for Estimate (B).

The conditional effect of carer subjective burden on anxiety symptoms was significant (*β* = .27, 95% CI [.05, .27], *p* = .006), as well as the conditional effect of experiential avoidance on anxiety symptoms (*β* = .44, 95% CI [.12, .35], *p* < .001). The interaction term (*β* = .21, 95% CI [.00, .02], *p* = .010) incrementally accounted for 3.4% of the variance in anxiety symptoms (*∆R*^*2*^ = .03). The total model explained 66% of the variance of anxiety symptomatology (*R*^*2*^ = .66, *F* (5,71) = 27.15, *p* < .001). The effect size for this regression model (Cohen’s *f*^2^) was 1.91, suggesting a large effect size. These results confirmed that generic experiential avoidance measured by the AAQ-II is a significant moderator of the relationship between carer subjective burden and anxiety symptoms.

**Figure 1.**
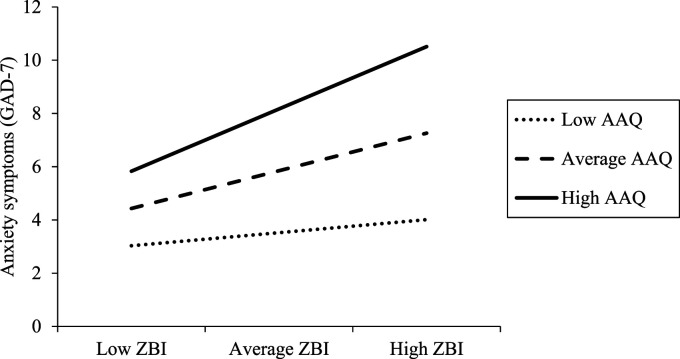
The interaction effect (carer subjective burden [ZBI] by experiential avoidance [AAQ]) in relation to anxiety symptoms.

In addition, the standardized slope for carer subjective burden regressed on anxiety symptoms was significant for individuals one *SD* above the mean level of the AAQ-II (High; B = .26, SE = .07, 95% CI [.11, .40], *p* = .001) and at the mean level of the AAQ-II (Average; B = .16, SE = .06, 95% CI [.05, .27], *p* = .006). However, the slope at one *SD* below the mean was not significant (Low; B = .05, SE = .06, 95% CI [−.07, .18], *p* = .389) (see Figure 1). When participants were presenting the same level of carer subjective burden, those who reported less experiential avoidance demonstrated fewer anxiety symptoms. This reverse buffering effect of experiential avoidance was stronger when individuals presented higher levels of carer subjective burden.

#### Experiential Avoidance in Caregiving (EACQ)

The results of the moderation analysis are shown in Table 4 with the unstandardized coefficients (*b*) and standard errors (SE) of the variables (independent variables [carer subjective burden]; moderator [experiential avoidance in caregiving]; interaction [carer subjective burden X experiential avoidance in caregiving]; and the two control variables [age and gender]).

**Table 4.** Results of Moderation Analysis - Experiential Avoidance in Caregiving (EACQ).

	β	Estimate (B)	SE	*t*	LLCI	ULCI	*p*
Intercept		7.01	3.88	1.81	−.73	14.74	.075
ZBI_centered (X)	.51	.30	.05	5.74	.19	.40	**<.001**
EACQ_centered (M)	.19	.12	.05	2.24	.01	.22	**.028**
ZBI x EACQ (XM)	.17	.01	.01	2.05	.00	.02	**.045**
Age (C_1_)	−.14	−.07	.04	−1.54	−.16	.02	.128
Gender (C_2_)	.17	2.04	1.05	1.95	−.04	4.13	.055
							
					*R*^2^ = .53, *MSE* = 14.06		
					*F* (5,71) = 15.87, *p* < .001		

*Notes.* ZBI, Zarit Burden Interview [assessment of carer subjective burden]; EACQ, Experiential Avoidance Caregiving Questionnaire [assessment of experiential avoidance related to caregiving]; LLCI, Lower level of 95% confidence interval for Estimate (B); ULCI, Upper level of 95% confidence interval for Estimate (B).

The conditional effects of both carer subjective burden (*β* = .51, 95% CI [.19, .40], *p* < .001) and experiential avoidance in caregiving (*β* = .19, 95% CI [.01, .22], *p* = .028) on anxiety symptoms were significant. The total model explained 53% of the variance of anxiety symptoms (*R*^*2*^ = .53, *F* (5,71) = 15.87, *p* < .001). This regression model showed a large effect size of 1.12 (Cohen’s f^2^). The addition of the interaction (*β* = .17, 95% CI [.00, .02], *p* = .045) did indicate a significant change beyond the main effect, incrementally accounting for 2.8% of the variance in anxiety symptomatology (*∆R*^*2*^ = .03). These results confirmed that experiential avoidance in caregiving assessed by the EACQ is a significant moderator of the relationship between carer subjective burden and anxiety symptoms.

**Figure 2.**
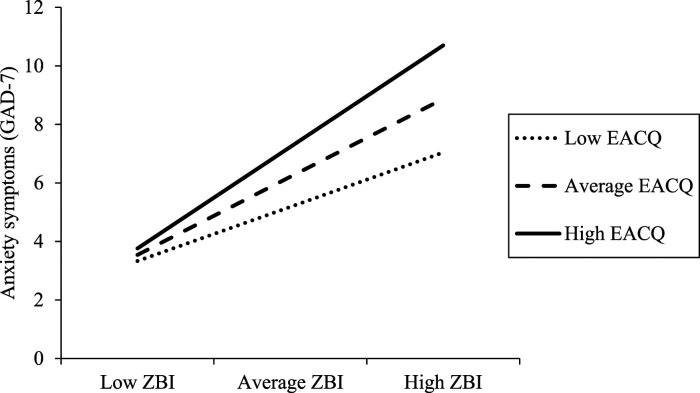
The interaction effect (carer subjective burden [ZBI] by experiential avoidance in caregiving [EACQ]) in relation to anxiety symptoms.

The standardized slope for carer subjective burden regressed on anxiety symptoms was significant for individuals one *SD* above the mean level of the EACQ (High; B = .38, SE = .07, 95% CI [.25, .52], *p* < .001), at the mean level of the EACQ (Average; B = .30, SE = .05, 95% CI [.19, .40], *p* < .001), and at one *SD* below the mean (Low; B = .21, SE = .07, 95% CI [.07, .34], *p* = .004) (see Figure 2). When participants were presenting the same level of carer subjective burden, those who demonstrated less experiential avoidance in caregiving reported fewer anxiety symptoms. The strength of this reverse buffering effect increased when participants presented higher levels of carer subjective burden.

## Discussion

Our results confirmed that both, generic experiential avoidance (measured by the AAQ-II) and experiential avoidance in caregiving (measured by the EACQ) were significantly moderating the relationship between carer subjective burden and anxiety symptoms. The strength of this reverse buffering effect increased when participants presented higher levels of carer subjective burden, suggesting that experiential avoidance could worsen the impact carer subjective burden has on anxiety symptoms, particularly among family carers experiencing higher levels of subjective burden.

Percentages of variance accounting beyond the main effects of experiential avoidance and carer subjective burden alone were significant in both models, with the interaction term of the AAQ-II and the EACQ accounting for 3.4% and 2.8% of the variance in anxiety symptoms, respectively. The interaction terms of the AAQ-II had a standardized coefficient of .208, whereas the standardized coefficient of the interaction term of the EACQ was .169. Although both interaction terms were statistically significant, there is potential that generic experiential avoidance may have a better moderating effect, which contradicts our hypothesis. This is also supported by visual inspection of slopes, which suggests that all slopes are steeper regardless of levels of the EACQ (Figure 2) compared to slopes for all levels of the AAQ-II (Figure 1).

Unlike the AAQ-II, which is the generic measure of experiential avoidance, the EACQ assesses experiential avoidance towards thoughts and feelings related to the person with dementia and the care provided to them (e.g., “I am scared of emotions and thoughts I have about my relative -with dementia-)” (Losada et al., 2014). Family carers are often faced with multiple stressors beyond their caregiving responsibilities such as their own physical health problems, financial difficulties, and lack of leisure time (Abdi et al., 2019; Lai, 2012). Therefore, family carers could be experiencing various unwanted internal experiences (e.g., worries about future disease progression or finances) and not simply distressing thoughts and feelings related to the person with dementia or the care provided to them. This could be specifically true for younger carers, who are more likely to deal with competing needs such as work demands (Liu et al., 2017). In these circumstances, the EACQ may be too narrow in their scope in capturing experiential avoidance presented by family carers.

The findings of this study provide important clinical implications in terms of assessment of experiential avoidance and interventions for preventing increased anxiety symptoms in family carers of people with dementia. Recent studies examining the association between carer subjective burden and Alzheimer’s disease severity and disease progression concluded that, as dementia progresses, carers are more likely to experience significant subjective burden (Froelich et al., 2021). This suggests that carers taking care of a person in the later stage of the disease may be more prone to higher levels of carer subjective burden. Since experiential avoidance moderates the relationship between carer subjective burden and anxiety symptoms; and this reverse buffering effect is likely to become more apparent as levels of burden increase (i.e., later stage of the disease), the development of early interventions for family carers of people with dementia are much needed. Recent studies found that acceptance and commitment therapy is effective for improving mental health problems among family carers of people with dementia (Fauth et al., 2022; Kishita et al., 2022). These interventions aim to reduce experiential avoidance by helping carers learn to step back from restricting thoughts and approach or allow painful emotions, while identifying personal values (what is most important to them) and engaging in such value-based activities (Hayes et al., 2013). Given the predictive difference of the measurements used in this study, monitoring the outcome of such interventions using the AAQ-II as the disease progresses is recommended.

While this study successfully examined the moderation effects of generic experiential avoidance and experiential avoidance in caregiving, there are some limitations that need consideration. First, this study employed the AAQ-II as a general measure of experiential avoidance. Although AAQ-II has been widely used in research, recent studies raised some concerns about its convergent and discriminant validity (Gámez et al., 2011; Vaughan-Johnston et al., 2017), highlighting that the concept measured by the AAQ-II may overlap with the underlying concept of the measures of psychological distress (Tyndall et al., 2019). Further studies may wish to consider using different measures of experiential avoidance such as the Multidimensional Experiential Avoidance Questionnaire (MEAQ; Gámez et al., 2011) to assess generic experiential avoidance in family carers of people with dementia and examine whether differences in findings can be found.

Considering the explained variances (66% for the AAQ-II and 53% for the EACQ), there may be other factors affecting the proposed moderation models. For example, a recent study examining the moderating role of experiential avoidance and cognitive fusion in a non-clinical sample concluded that experiential avoidance indirectly contributed to depression and anxiety through cognitive fusion rather than experiential avoidance directly affecting depression and anxiety (Cookson et al., 2020). Cognitive fusion refers to our tendency to become entangled with thoughts and inability to step back from such restricting beliefs (Hayes et al., 2013). Future studies could explore such combined moderating effect of experiential avoidance and cognitive fusion on the relationship between stressors and mental health outcomes in family carers of people with dementia.

The current study collected data during the COVID-19 pandemic; therefore, results may be affected by the additional stress the pandemic brought to carers (Rising et al., 2022). In addition, this study did not collect information on the ethnicity of participants. Participants in this study were recruited in counties in the east of England where more than 90% of the population is White British, potentially resulting in a non-diverse sample. In addition, participants were mainly female, and half of the participants experienced minimal to mild anxiety symptoms, which may limit the generalizability of the findings. Future studies should therefore investigate a wider population and include male family carers, those from different ethnic backgrounds and a clinical population (i.e., participants with more severe anxiety symptoms).

Finally, the sample size required for a regression model to achieve a power level of .80, a significance level of .05 and a medium effect size (.15) is 92 when five independent variables are used. This study had a sample size of 77, which is smaller than required although the effect size for both regression models was large in this study. The cross-sectional nature of this study also does not allow for any causal assumptions to be made, and thus, the findings need to be interpreted with caution. In conclusion, it is recommended that future studies replicate this study in family carers of people with dementia with higher levels of anxiety, using different measures of generic experiential avoidance (e.g., MEAQ) alongside the AAQ-II and in a longitudinal design.

Despite limitations, this study provided evidence supporting that experiential avoidance may enhance the negative effect of carer subjective burden on anxiety symptoms in family carers of people with dementia. The strength of this reverse buffering effect seems to increase when carers present higher levels of subjective burden. The development of early interventions aimed at undermining experiential avoidance and monitoring the outcomes using the AAQ-II as the disease progresses may be beneficial for preventing increased anxiety symptoms among this population.
